# Supplementary material for: Synaptic organization of the Drosophila antennal lobe and its regulation by the Teneurins
Source: eLife. 2014 Oct 13;3:e03726. doi: 10.7554/eLife.03726 (PMC4194450; doi:10.7554/eLife.03726)
Supplement: Figure 2—source data 1. — Table of statistical values for three potential sum of Gaussian fits each for the DL4 and DM6 MARCM datasets. DOI: http://dx.doi.org/10.7554/eLife.03726.008 [file elife03726s001.docx]

Source Data 1 for Figure 2: Mosca and Luo

Table of Gaussian Curve-Fitting Data

| Glomerulus | Terms | SSE | *r^2^* | RMSE | AICc | F-test (*p* value) | |
| --- | --- | --- | --- | --- | --- | --- | --- |
|  |  |  |  |  |  |  |  |
| DL4 | 3 | 146.3 | 0.592 | 2.372 | +47.09 | 0.0001^a^ |  |
| DL4 | 4 | 35.13 | 0.902 | 1.236 | -91.93 |  | 0.3471^b^ |
| DL4 | 6 | 34.34 | 0.904 | 1.421 | -89.58 |  |  |
|  |  |  |  |  |  |  |  |
| DM6 | 3 | 107.0 | 0.642 | 2.029 | +16.12 | 0.0001^c^ |  |
| DM6 | 4 | 62.85 | 0.790 | 1.653 | -34.34 |  | 0.0001^d^ |
| DM6 | 5 | 24.28 | 0.919 | 1.107 | -126.25 |  |  |
|  |  |  |  |  |  |  |  |
|  |  |  |  |  |  |  |  |

^a^ Comparing the 3-term and 4-term DL4 models; *H_0_* = 3-term is true

^b^ Comparing the 4-term and 6-term DL4 models; *H_0_* = 4-term is true

^c^ Comparing the 3-term and 4-term DM6 models; *H_0_* = 3-term is true

^d^ Comparing the 4-term and 5-term DM6 models; *H_0_* = 4-term is true

SSE = Sum of Squared Errors of Prediction

RMSE = Root Mean Square Error

AICc = Akaike’s Information Criteria (corrected)
