# Supplementary material for: Synaptic organization of the Drosophila antennal lobe and its regulation by the Teneurins
Source: eLife. 2014 Oct 13;3:e03726. doi: 10.7554/eLife.03726 (PMC4194450; doi:10.7554/eLife.03726)
Supplement: Figure 2—source data 2. — Table of simulation data for three distributions of the DL4 and DM6 MARCM data sets and the probabilities of obtaining simulated data more evenly distributed than observed. DOI: http://dx.doi.org/10.7554/eLife.03726.009 [file elife03726s002.docx]

Source Data 2 for Figure 2: Mosca and Luo

Table of Resampling Information

| Glomerulus | Distribution Fit | No. of Simulations | Simulations with *S* < Observed | Probability of  *S* < Observed |
| --- | --- | --- | --- | --- |
|  |  |  |  |  |
| DL4 | Gaussian | 50000 | 0 | 0.00000 |
| DL4 | Poisson | 50000 | 24 | 0.00048 |
| DL4 | Uniform | 50000 | 3 | 0.00006 |
|  |  |  |  |  |
| DM6 | Gaussian | 50000 | 30 | 0.00060 |
| DM6 | Poisson | 50000 | 1333 | 0.02666 |
| DM6 | Uniform | 50000 | 42 | 0.00084 |
|  |  |  |  |  |

Three different distributions were used to model the aggregate data; of these the Poisson distribution yielded the highest probability of peaks within the simulated data being as evenly spaced or more than the observed data. Thus, we present these values as the most conservative estimate of probability, which still fall below the 0.05 confidence level.

Here, S = σ {(μ_1_-μ_2_), (μ_2_-μ_3_), (μ_3_-μ_4_)}
